# Calpain inhibition as a possible new therapeutic target in multiple sclerosis

**DOI:** 10.3934/molsci.2017.4.446

**Published:** 2017-10-26

**Authors:** Amena W. Smith, Swapan K. Ray, Arabinda Das, Kenkichi Nozaki, Baerbel Rohrer, Naren L. Banik

**Affiliations:** 1 Department of Neurosciences, Medical University of South Carolina, Charleston, SC, USA; 2 Department of Pathology, Microbiology and Immunology, University of South Carolina, Columbia, SC, USA; 3 Department of Neurosurgery, Medical University of South Carolina, Charleston, SC, USA; 4 Department of Ophthalmology, Medical University of South Carolina, Charleston, SC, USA; 5 Research Service, Ralph H. Johnson VA Medical Center, Charleston, SC, USA

**Keywords:** inflammation, demyelination, neurodegeneration, oligodendrocytes, neurons, retinal ganglion cells, axonal degeneration, optic neuritis, calpain, calpain inhibition

## Abstract

Multiple sclerosis (MS), the most common chronic autoimmune inflammatory disease of the central nervous system (CNS), is characterized by demyelination and neurodegeneration. In particular, neurodegeneration is a major factor in disease progression with neuronal death and irreversible axonal damage leading to disability. MS is manageable with current therapies that are directed towards immunomodulation but there are no available therapies for neuroprotection. The complex pathophysiology and heterogeneity of MS indicate that therapeutic agents should be directed to both the inflammatory and neurodegenerative arms of the disease. Activity of the Ca^2+^ activated protease calpain has been previously implicated in progression of MS and its primary animal model, experimental autoimmune encephalomyelitis (EAE). The effects of calpain inhibitors in EAE involve downregulation of Th1/Th17 inflammatory responses and promotion of regulatory T cells, overall leading to decreased inflammatory cell infiltration in CNS tissues. Furthermore, analysis of brains, spinal cords and optic nerves from EAE animals revealed decreases in axon degeneration, motor neuron and retinal ganglion cell death. This resulted in improved severity of paralysis and preservation of visual function. Taken together, the studies presented in this brief review suggest that use of calpain inhibitors in combination with an immunomodulatory agent may be a potential therapeutic strategy for MS and optic neuritis.

## 1. Introduction

### 1.1. Epidemiology of MS

Multiple Sclerosis (MS) is an autoimmune inflammatory and neurodegenerative disease of the central nervous system (CNS) that is the leading cause of neurologic disability in young adulthood, and there is no cure for the disease [1]. The worldwide disease burden of MS is upwards of 2.5 million individuals, with an incidence of approximately 7 new cases per 100,000 people per year and a lifetime risk of 1 in 400. An estimated 400,000 cases have been diagnosed in the US with roughly 200 new cases every week. As many as 26% of MS patients live without impaired mobility in their lifetime, but a minority (15%) become rapidly disabled [2,3]. The reduction in life expectancy due to MS is small, so most patients live at least 25 years after diagnosis, and the majority experience increasing neurologic disability for the duration of their lives. Estimates suggest that MS results in $9.5 billion in medical costs and lost productivity each year.

MS was first described nearly 200 years ago but many questions about causal factors, individual susceptibility and biomarkers of disease progression remain [4,5]. Current treatments are directed towards immunomodulation but do not specifically target neurodegenerative processes. As such, therapies need to be developed that are directed towards this aspect of the disease, especially since damage to axons and neurons appears to predict long term disability in MS [4,6–8].

### 1.2. Pathophysiology and Clinically Isolated Syndrome of Multiple Sclerosis

The current consensus is that MS pathology can be attributed to an inflammatory response subsequent to activation of auto-reactive T cells specific for myelin antigens. While the exact mechanisms of MS induction are still unknown, epitope mimicry and disturbed immune surveillance are the most well-studied theories of how infection may induce MS in susceptible individuals [9]. Within the concept of epitope mimicry, one becomes infected by a virus or bacterium that expresses a protein with a sequence very similar to that of a CNS self-protein. The infection thus indirectly triggers an autoaggressive lymphocyte response that leads to progression of MS [10]. There is also increasing evidence that the CNS is continuously surveyed by virus-specific T cells, which protect against reactivating neurotropic viruses. Acute viral infections may lead to the breakdown of self-tolerance in genetically predisposed individuals, resulting in reactivations of viruses in the CNS that induce the recruitment of both autoaggressive and virus-specific T cell subsets, causing relapses and cumulative disability [11].

The monitoring of a potential MS case begins when patients present with a Clinically Isolated Syndrome (CIS), which is a single neurologic attack compatible with MS. CIS commonly presents as difficulty walking, pain or numbness of the lower extremities, loss of central vision, double vision, bladder dysfunction, or cognitive deficits [3,12]. Studies in CIS patients suggest that gray matter atrophy occurs very early in the MS disease course [13]. Moreover, cerebral atrophy may even begin well before the clinical symptoms appear, and it may be independent of cortical demyelination. Klaver et al. found neurodegenerative changes in both normally myelinated normal-appearing gray matter (NAGM) and Type III MS lesions, however there was no significant difference in neuron and axon density between NAGM and Type III MS lesions [14]. De Stefano demonstrated the presence of widespread, irreversible axonal damage independent of inflammatory lesions in the cerebral white matter at very early stages of the disease and despite heterogeneity of MS subtype [15]. One pathological mechanism of gray matter atrophy is damage secondary to demyelination and axonal transection within an inflammatory lesion [16], but this mechanism alone is insufficient to explain why atrophy is at times scarce in the presence of inflammation and at times significant in regions with little or no active inflammatory demyelination [17,18]. Thus, the task of elucidating the molecular mechanisms of the independent neurodegenerative process is at hand.

### 1.3. Optic neuritis

Optic neuritis (ON) is strongly associated with multiple sclerosis (MS) and is the first sign in the diagnosis of 15–20% of MS cases. Clinical electrophysiology and optical coherence tomography studies have demonstrated degeneration of the optic nerve and retina following an optic neuritis flare [19]. Corollary animal studies have shown oligodendroglial death, myelin and axonal damage, and retinal ganglion cell (RGC) death [20]. These events herald acute loss of visual acuity, contrast sensitivity, and visual field defects that are not fully reversible due to residual axon damage and neuronal loss [21–24]. Changes to visual pathway structure in MS have been described as a window into the CNS, yielding valuable information about larger disease processes.

Current approved disease-modifying therapies (DMTs), based on their mechanisms of action, are limited in their ability to protect or repair damage to the optic nerve, brain and spinal cord. Addition of a neuroprotective agent to the DMT regimen may significantly delay disease progression and provide an option for patients who receive minimal benefit from DMT alone.

MS and ON have been well-studied in the animal model experimental autoimmune encephalomyelitis (EAE) [25–27]. Increased expression and activity of calpain, a Ca^2+^-activated protease, has been detected in the CNS tissues of EAE rodents during disease exacerbations. Further study has revealed that activated calpain plays a role in promotion of inflammation, demyelination, and neurodegeneration in MS disease models [28–30]. Calcium influx and calpain activation early in the disease course are hypothesized to contribute to neurological impairment in EAE and MS. Therefore, prophylactic or therapeutic treatment with calpain inhibitor may attenuate the events that lead to cell death and axonal damage in these diseases, ultimately improving neurological function.

### 1.4. Calpain

Among the proteolytic enzymes, calpain stands out as an important player in the pathogenesis of many CNS disorders, including traumatic brain injury [31], spinal cord injury [32], Parkinson’s Disease and MS [33–35]. Calpain, a Ca^2+^-activated cysteine protease, is a neutral and non-lysosomal proteolytic enzyme typically found in the cytosol of all animal cells. Inactive calpain is a pro-enzyme that is activated in the cytosol with an increase in intracellular free Ca^2+^ concentrations. The pro-enzyme may also relocate to the membrane for activation in contact with membrane-bound phospholipids and then for degradation of membrane proteins. Fifteen genes within the human genome encode the calpain isoforms that form the calpain family [36]. Some of the calpain isoforms are found in specific tissues. Only two major calpain isoforms, μ-calpain, and m-calpain, are expressed in all cell types. In this review “calpain” refers to the ubiquitous calpain isoforms, μ-calpain and m-calpain, which require 2–80 μM and 0.2–0.8 mM Ca^2+^ concentrations, respectively, for half-maximal activity *in vitro*. The μ-calpain and m-calpain isoforms exist as heterodimers consisting of a catalytic 80 kD large subunit and a regulatory 30 kD small subunit. Calpain exists as a pro-enzyme in the cytosol where the normal range of free Ca^2+^ concentration is 50–100 nM in resting cells [37]. An increase in the cytoplasmic free Ca^2+^ concentration activates calpain, producing the active 76 kD fragment for proteolysis in the cells. At the normal range of intracellular free Ca^2+^ concentrations, calpain is activated to perform important physiological functions such as cytoskeletal rearrangement, long-term potentiation, processing of hormones, and protein turnover [38]. However, when intracellular free Ca^2+^ concentrations exceed the normal threshold, uncontrolled and prolonged calpain activation may occur. An increased activation of ubiquitous calpain has been associated with processes integral to demyelination and neurodegeneration [31,39,40].

Both μ-calpain and m-calpain demonstrate the same substrate specificity. Cytoskeletal proteins, membrane proteins, cytokines, transcription factors, protein kinases, phosphatases, and lens proteins are preferred calpain substrates. Calpain substrates are proteolyzed in a limited manner rather than digested to smaller peptides, suggesting a modulation of their function [41]. Calpastatin is an endogenous protein inhibitor that specifically inhibits ubiquitous calpain activity [36]. The increase of the calpain/calpastatin ratio is a consequence of excessive activation of calpain that can degrade calpastatin either in a conservative or non-conservative way. Calpastatin is always considered a suicide substrate, but in normal conditions of calpain activity, this ratio, instead of increasing, decreases as conservative calpastatin digestion produces units retaining the ability to inhibit calpain [36]. Calpastatin is not cell permeable, and therefore is not usable as a therapeutic option for inhibition of intracellular calpain activity. Extensive research has, however, produced different groups of cell-permeable calpain inhibitors, some of which appear to be promising for neuroprotection in animal models of CNS injuries and diseases such as acute viral encephalitis [42], stroke and Parkinson’s Disease [43–44].

## 2. Synthetic calpain inhibitors

### 2.1. Calpeptin

Several commercially available calpain inhibitors are employed in experimental studies of MS. A challenging aspect of calpain inhibitor development has been to identify calpain-selective inhibitors, as many older generation agents also inhibit other cysteine proteases, serine proteases or the proteosome [45]. Synthetic calpain inhibitors can be categorized into two major groups, the peptide and non-peptide inhibitors. The peptide inhibitors can be further classified as reversible (peptidyl aldehydes, peptidyl α-ketoamides) or irreversible inhibitors (peptidyl epoxides) [46]. However, the peptide inhibitors have a common mechanism of action that involves formation of hemiacetal or theohemiacetals with active site cysteine or serine residues. Since μ and m-calpain are ubiquitously expressed and have diverse physiological functions, reversible inhibitors are preferred in order to avoid side effects secondary to permanent, non-specific blockage of calpain activity. Furthermore, the cellular permeability of these inhibitors has been enhanced via N-terminal capping and esterification of the carboxyl group with lipophilic groups [47,48]. Peptidyl epoxides and peptidyl aldehydes in particular have greater selectivity for cysteine proteases vs. serine proteases [49,50]. Calpeptin (EMD Biochemicals, Gibbstown, NJ) is a cell-permeable peptidyl aldehyde inhibitor of μ and m-calpain. Although any protease with active site cysteine residues (e.g. papain) may be targeted, calpeptin is highly substrate selective for calpains 1 and 2 (ID_50_ = 52 nM for calpain-1; ID_50_ = 34 nM for calpain-2; ID_50_ = 138 nM for papain). Calpeptin has been shown to prevent apoptosis of various cell types in vitro, including lympho cytes [51], cardio myocytes [52–54], primary oligodendrocytes [55], and retinal ganglion cells (RGCs) [56]. Furthermore, calpeptin treatment can reduce the development of acute and chronic inflammation *in vivo* and attenuate signs of EAE paralysis [57,58]. A limitation of calpeptin for in vivo use is its poor aqueous solubility, which necessitates intraperitoneal delivery of this agent after it has been dissolved in dimethyl sulfoxide (DMSO).

Peptidyl ketoamides comprise a third generation of calpain inhibitors that are characterized by improved potency, cell permeability and high selectivity for calpains relative to other cysteine proteases. These include morpholines (e.g. AK275, AK295), chromones, and benzodioxothianzines (SJA-6017 derivatives) [59]. Oral delivery is preferred over injectible delivery for the treatment of chronic CNS diseases such as MS. Oral administration of SJA 6017 has curtailed neuronal cell death in animal models of ischemia, cataracts, traumatic brain injury (TBI), photoreceptor degeneration, and glutamate excitotoxicity, indicating that the inhibitors cross the BBB and are cell permeable [46,56,60–62]. However, high doses have been required to achieve neuroprotective effects since SJA 6017 has poor oral bioavailability due to extensive metabolism of its aldehyde group [46–63]. SNJ 1945, a hemiacetal derivative of SJA 6017, is improved in terms of potency, aqueous solubility, oral bioavailability, and extended plasma half-life in rodent models of cerebral ischemia and retinal ganglion cell (RGC) damage [63–66]. The non-peptidyl calpain inhibitors constitute the most recent generation of calpain inhibitors. These reversible non-competitive inhibitors interact with calpain domains relevant for their activation, are characterized by superior stability and selectivity than the peptidyl inhibitors, and have thus far demonstrated potent protection of cultured neurons [46,63].

### 2.2. A role for calpain in inflammatory demyelinating disease

As mentioned previously, the hallmark features of MS and EAE are inflammation, demyelination, and neurodegeneration. In various experimental settings, calpain activity has been shown to play a role in all three pathophysiological events (Table 1). Calpain activity plays a key role in increasing the expression of many pro-inflammatory mediators (e.g. NF-κB, Cox-2) and participates in activation and migration of T cells [67–71]. Calpain released from activated T cells degrades myelin basic protein (MBP) and other myelin components in vitro and in a rat model of prenatal hypoxia-ischemia [72,73]. Calpain activity has also been implicated in axonal damage and death of neurons and oligodendrocytes [35,39,74,75], at least partially through modulation of proteins involved in classical receptor and mitochondrial apoptotic pathways [76–78]. Specifically, in a Lewis rat model of acute EAE [77], an increase in TUNEL-positive neurons and internucleosomal DNA fragmentation in EAE spinal cords suggested that neurons died by apoptosis on days 8–10 following EAE induction. Beginning on day 8 of EAE, increases in calpain expression in the spinal cord correlated with activation of pro-apoptotic proteases, which occurred prior to the appearance of paralysis. An increase in calcineurin expression and a decrease in phospho-Bad (p-Bad) suggested that Bad activation contributed to apoptosis during acute EAE. An increase in the Bax: Bcl-2 ratio and activation of caspase-9 indicated involvement of the mitochondria in apoptosis. Caspase-8 activation suggested induction of the death receptor-mediated pathway of apoptosis. Endoplasmic reticulum stress leading to caspase-3 activation was also observed, leading to the conclusion that multiple apoptotic pathways were activated in the spinal cord during acute EAE, and that activated calpain has a role in neuronal apoptosis during the development of acute EAE. To test the hypothesis that calpain inhibition can alleviate disability in EAE, two experimental models were utilized. First, treatment of actively immunized EAE Lewis rats with the calpain inhibitor calpeptin (50–250 microg/kg) dose-dependently ameliorated paralysis [77]. In the second model, MBP-specific T cells were harvested from actively immunized SJL/J mice and were then incubated in the presence or absence of the calpain inhibitor SJA6017 prior to adoptive transfer (AT) of the T cells to naive SJL/J mice. EAE mice that received MBP-specific T cells incubated with SJA6017 before AT demonstrated a dose-dependent reduction in the degree of paralysis during relapses as well as a reduction in the frequency of relapses [57]. In both models, calpain activity, astrocyte reactivity, loss of myelin, axonal damage, and neuronal and oligodendrocyte death were attenuated in the spinal cord with calpain inhibitor therapy. Thus, the advantage of calpain inhibitors in comparison to the majority of current therapeutic agents is that they target both neurodegenerative and inflammatory pathways of disease in EAE.

In addition to experimental evidence of a role for calpain in inflammatory demyelinating disease, clinical data from MS patients is emerging. Expression and activity of calpain was increased in post-mortem brain tissue from MS patients and has been co-localized with glial and CD4+ T cells and damaged axons in MS plaques, which has also been seen in EAE [39,99,100]. The activation of myelin-specific T cells is thought to be a major event in the development and progression of MS and its models. Production of pro-inflammatory Th1 cytokines (e.g. TNFα, IL-2, and IFNγ) by CD4^+^ T cells increases during an MS exacerbation [101,102]. In contrast, anti-inflammatory Th2 cytokines (e.g. IL-4, IL-10, IL-13) are predominant during disease remission [103–105]. Th17 cells also contribute to autoimmune pathogenesis, and cytokines that promote their development and proliferation include IL-17, IL-23, and IL-6 [106–108]. Th17 cells can efficiently cross the blood-brain barrier using alternate paths from Th1 cells, promote blood brain barrier disruption, and induce activation of other inflammatory cells in the CNS [109]. Furthermore, increased numbers of peripheral blood mononuclear cells including CD4+ and CD8+ T cells have been shown to express high levels of IL-17 mRNA during MS relapses [107,110,111]. On the other hand, dendritic cells (DCs) of MS patients and EAE mice express abnormally low levels of cytoplasmic indoleamine 2,3-dioxygenase (IDO) [112]. IDO is a heme-containing enzyme that catalyzes the aerobic metabolism of L-tryptophan to N-formylkynurenine, which is the first and rate-limiting step in the kynurenine pathway. Initiation of the IDO-kynurenine pathway facilitates immune inhibitory function, and local DO-mediated tryptophan depletion leads to starvation and stress of Th1 cells, impaired function of bystander Th1 cells, and apoptosis [113–116]. Therefore, a high level of IDO expression may precede a favorable shift in Th1/Th2-mediated immune responses in chronic inflammatory diseases such as MS, and measurement of IDO gene expression and activity in the blood is being explored as a useful biomarker to monitor the course of relapsing-remiting MS [117,118]. Higher levels of calpain are expressed in both unactivated and activated PBMCs from MS patients compared to controls, and inhibition of calpain in these cells attenuates secretion of IL-2 and IFNγ, which may promote an anti-inflammatory cytokine bias [89]. Furthermore, calpain inhibition in primary myelin basic protein-specific T cell cultures increases Th2 proliferation and cytokine profile with relative decreases in Th1 and Th17 proliferation [66]. Further investigation demonstrated that calpain inhibition downregulated several pro-inflammatory cytokines (IL-17, IL-23, TNFα, G-CSF, IL-12) in MS PBMCs while it upregulated IDO expression and limited T cell proliferation, indicating that inhibition of calpain may ameliorate immune pathology in MS [119].

### 2.3. The efficacy of calpain inhibition in optic neuritis

The optic nerve serves as a marker of neurodegeneration for other CNS tissues in MS and EAE [120]. The visual dysfunction that accompanies ON is a common cause of disability and reduced quality of life in MS [121,122]. Intravenous methylprednisolone (MP) is currently the drug of choice to hasten visual recovery and reduce the risk of MS during an acute episode of ON. However, MP fails to improve long-term visual disability [22,123]. A prospective cohort study of EAE mice revealed that disruption of the blood-optic nerve barrier (BONB) began as early as 3 days post-immunization [77], suggesting that ON may be an early event during EAE progression. As observed in MS patients, EAE-ON animals also demonstrate pathological changes in visual evoked potential (VEP) and electroretinogram (ERG) recordings, which reflect damage to the optic nerve and retina, respectively [26,67,124]. Increased expression and activity of μ-calpain and m-calpain has been detected in EAE-ON optic nerves prior to paralysis onset [125,126]. Moreover, treatment with EAE rats with calpeptin reduced the level of active calpain expression and attenuated inflammatory marker expression (iNOS, COX-2, NF-kB), microgliosis, astrogliosis, and expression of aquaporin 4 (AQP4). The Bax: Bcl-2 ratio, production of tBid, PARP-1, expression and activities of pro-apoptotic caspases, and internucleosomal DNA fragmentation were also attenuated in the optic nerves of treated rats [126]. In a related EAE study, calpeptin was shown to prevent apoptosis of retinal ganglion cells (RGCs) by downregulating expression of pro-apoptotic proteins and the pro-inflammatory molecule nuclear factor-kappa B (NF-κB) [74,119,126]. Furthermore, a rat model of EAE induced with myelin oligodendrocyte glycoprotein demonstrated that significant RGC loss precedes the onset of pathologically defined ON, and calpeptin attenuates loss of RGCs [35,127]. In the study by Hoffman et al. [127], manganese-enhanced magnetic resonance imaging was used to monitor preclinical calcium elevations in the retina and optic nerve of induced rats. Calcium elevation correlated with an increase in calpain activation during the induction phase of ON, as revealed by increased calpain-specific cleavage of spectrin. Calpeptin treatment reduced calpain activity and protected retinal ganglion cells from degeneration. These results further establish that elevation of retinal calcium levels and calpain activation are early events in ON, which make this pathway an ideal target for therapeutic intervention.

The neuroprotective efficacy of SNJ 1945, an inhibitor with superior oral bioavailability to calpeptin, has mainly been tested in mouse models of retinal disease. In a model of N-methyl D-aspartate (NMDA)-induced excitotoxic injury, two oral doses of SNJ 1945 significantly inhibited TUNEL+ cell death in the ganglion cell layer (GCL) and inner nuclear layer (INL) and prevented thinning of the inner photoreceptor layer (IPL). Furthermore, levels of cleaved spectrin fragments (surrogates of calpain activity) were increased as early as 6 h after NMDA injection, and spectrin cleavage was attenuated by SNJ 1945 [128]. Similarly, oral dosing of SNJ 1945 prevented outer nuclear layer (ONL) atrophy in a mouse model of light-induced retinal degeneration [129]. An in vivo mouse model of oxidative stress in RGCs showed that oxidative stress directly activated the calpain pathway and induced RGC death. Importantly, inhbition of the calpain pathway with SNJ 1945 was neuroprotective [130]. In an ON treatment study, EAE was induced in B10.PL mice with myelin basic protein at Day 0, then the mice received twice daily oral dosing of SNJ 1945 from Day 9 until sacrifice on Day 26 [35]. Throughout the study, visual function was determined by electroretinogram recordings and daily measurement of optokinetic responses (OKR) to a changing pattern stimulus. EAE mice manifested losses in OKR thresholds, a measurement of visual acuity, which began early in the disease course. There was a significant bias toward unilateral visual impairment among EAE-ON eyes. Treatment with SNJ 1945, initiated after the onset of OKR threshold decline, improved visual acuity and pattern electroretinogram amplitudes, with attenuation of RGC death. Furthermore, calpain inhibition spared oligodendrocytes, prevented degradation of axonal neurofilament protein, and attenuated reactive astrocytosis. The finding of early, unilateral visual impairment in this mouse model parallels the clinical presentation of ON. Overall, these findings suggest that administration of an orally bioavailable calpain inhibitor may preserve visual function in clinical ON.

## 3. Future Studies

Some of the *in vitro* studies described above can be enhanced by the introduction of calpain 1 or 2 gene silencing in certain cell types, which allow unequivocal testing of a role for calpain in immune and neurodegenerative processes [63]. Use of silencing might aid the identification of a molecular mechanism for events such as upregulation of IDO. Moreover, a calpain heterozygous transgenic mouse and a calpastatin knockout mouse model are being used in related studies of neuroprotection and utilization of these animals could greatly enhance the understanding of EAE pathophysiology and provide a standard by which the efficacy of inhibitors can be compared [131–133].

Although the neuroprotective efficacy of calpain inhibitors has been established, their use in chronic demyelinating disease is limited by their ability to cross the blood-brain barrier. Fusion of calpain inhibitors with molecules that have specific transporters in the brain (e.g. dendrimers) may be an alternative strategy to achieve entry into the CNS that also allows dose-sparing [134,135].

Finally, an important theme that has emerged is the concept of pairing a neuroprotectant compound with an established disease-modifying therapy to treat EAE or MS. Data from a recent report indicates that natalizumab treatment can actually reduce the accumulation of nerve injury in relapsing-remitting multiple sclerosis, as assessed by release of NFL into the CSF [136]. Likewise, in EAE, IFN-β-1a treatment three times per week slightly decreases the loss of RGCs. However, in contrast to neurotrophic factor effects, IFN-β-1a does not directly protect cultured RGCs from apoptosis. IFN-β-1a was found to be a suitable candidate to be combined with a directly neuroprotective agent such as a calpain inhibitor in order to further decrease axonal and neuronal degeneration in MS patients [137].

## 4. Conclusion

As re-iterated throughout this review, MS and ON have inflammatory, demyelinating, and neurodegenerative aspects that must be further elucidated and addressed therapeutically. More importantly, although patients experience disease relapses and remissions, gray matter damage is permanent and appears to be strongly associated with disability progression. Previous studies have highlighted the fact that known mechanisms of axon degeneration, namely, impaired axonal transport from the cell body, mitochondrial failure, and an increase in intra-axonal calcium, involve calpain-mediated degradation of axonal proteins [138]. The studies presented in this review build a case that calpain inhibition, possibly in combination with an approved immunomodulatory agent, is a potential novel strategy for neuroprotection in MS.

## Figures and Tables

**Table 1.** Roles and Substrates of Active Calpain in Inflammatory Demyelinating Disease

Inflammation	T cell activation Cleaves the NfκB inhibitor IkBa → expression of NFkB-dependent genes (e.g. iNOS, COX-2, IL-2, CD25) [78,80] Activates STAT 3, STAT5 → pro and anti-inflammatory cytokine production [31,81] Inactivates STAT6 → ↓ anti-inflammatory IL-4 levels to prevent differentiation of naïve CD4+ T cells to Th2 subtype [82–84]
	T cell migration Calpain modulates the LFA signaling pathway in migrating T lymphocytes [85–87] CI prevented chemotaxis of T cells toward CCL2 chemokine [69] Calpain activity correlates with T cell and macrophage migration into the CNS during EAE [31]
Demyelination	Degrades MBP in primary oligodendrocyte culture upon acute Ca^2+^ influx [55] Degrades MBP after PTM (e.g. citrullination) → emergence of immunogenic epitopes in MS [88,89]
Neurodegeneration	Degrades axonal proteins (e.g. NFP) [28,90,91] Alters cytoskeletal proteins [92–94]
Cell Death	Apoptosis of glia and neurons in neurodegenerative diseases [66,95–98]

STAT (signal transducer and activator of signaling); CI (calpain inhibitor); LFA (leukocyte function-associated antigen); MBP (myelin basic protein); PTM (post-translational modification); NFP (neurofilament protein)

## References

[1] Dargahi N, Katsara M, Tselios T, (2017) Multiple Sclerosis: Immunopathology and Treatment Update. Brain Sci 7: 78. 28686222 10.3390/brainsci7070078 PMC5532591

[2] Confavreux C, Vukusic S (2014) The clinical course of multiple sclerosis. Handb Clin Neurol 122: 343–369. 24507525 10.1016/B978-0-444-52001-2.00014-5

[3] Confavreux C, Vukusic S, Moreau T, (2001) Relapses and progression of disability in multiple sclerosis. N Engl J Med 343: 1430–1438. 10.1056/NEJM200011163432001 11078767

[4] Kinzel S, Weber MS (2016) B Cell-Directed Therapeutics in Multiple Sclerosis: Rationale and Clinical Evidence. Cns Drug 30: 1137–1148. 10.1007/s40263-016-0396-6 27844213

[5] Fernando NT, Koch M, Rothrock C, (2008) Tumor escape from endogenous, extracellular matrix-associated angiogenesis inhibitors by up-regulation of multiple proangiogenic factors. Clin Cancer Res 14: 1529–1539. 18316578 10.1158/1078-0432.CCR-07-4126

[6] Rottlaender A, Kuerten S. (2015) Stepchild or Prodigy? Neuroprotection in Multiple Sclerosis (MS) Research. Int J Mol Sci 16: 14850–14865. 26140377 10.3390/ijms160714850 PMC4519875

[7] Popescu V, Agosta F, Hulst HE, (2013) Brain atrophy and lesion load predict long term disability in multiple sclerosis. J Neurol Neurosur Psychiatr 84: 1082–1091. 10.1136/jnnp-2012-304094 23524331

[8] Vickers JC, King AE, Woodhouse A, (2009) Axonopathy and cytoskeletal disruption in degenerative diseases of the central nervous system. Brain Res Bull 80: 217–223. 19683034 10.1016/j.brainresbull.2009.08.004

[9] Mentis AA, Dardiotis E, Grigoriadis N, (2017) Viruses and Multiple Sclerosis: From Mechanisms and Pathways to Translational Research Opportunities. Mol neurobiol 54: 3911–3923. 28455696 10.1007/s12035-017-0530-6

[10] Berer K, Krishnamoorthy G (2014) Microbial view of central nervous system autoimmunity. FEBS lett 588: 4207–4213. 24746689 10.1016/j.febslet.2014.04.007

[11] Geginat J, Paroni M, Pagani M, (2017) The Enigmatic Role of Viruses in Multiple Sclerosis: Molecular Mimicry or Disturbed Immune Surveillance? Trend in Immunol 38: 498–512. 10.1016/j.it.2017.04.006 PMC7185415 28549714

[12] Brownlee WJ, Miller DH (2014) Clinically isolated syndromes and the relationship to multiple sclerosis. J Clin Neurosci 21: 2065–2071. 25027666 10.1016/j.jocn.2014.02.026

[13] Rocca MA, Preziosa P, Mesaros S, (2016) Clinically Isolated Syndrome Suggestive of Multiple Sclerosis: Dynamic Patterns of Gray and White Matter Changes-A 2-year MR Imaging Study. Radiology 278: 841–853. 26348234 10.1148/radiol.2015150532

[14] Klaver R, Popescu V, Voorn P, (2015) Neuronal and axonal loss in normal-appearing gray matter and subpial lesions in multiple sclerosis. J Neuropathol Exp Neurol 74: 453–458. 25853695 10.1097/NEN.0000000000000189

[15] De SN, Giorgio A, Battaglini M, (2010) Assessing brain atrophy rates in a large population of untreated multiple sclerosis subtypes. Neurology 74: 1868–1876. 20530323 10.1212/WNL.0b013e3181e24136

[16] Alonso A, Hernán MA (2008) Temporal trends in the incidence of multiple sclerosis: a systematic review. Neurology 71: 129–135. 18606967 10.1212/01.wnl.0000316802.35974.34 PMC4109189

[17] Rojas JI, Patrucco L, Miguez J, (2016) Brain atrophy in multiple sclerosis: therapeutic, cognitive and clinical impact. Arq Neuro-Psiquiat 74: 235–243. 10.1590/0004-282X20160015 27050854

[18] Rojas JI, Patrucco L, Besada C, (2010) [Brain atrophy in clinically isolated syndrome]. Neurología 25: 430–434. 20964989

[19] Tawse KL, Gobuty M, Mendoza-Santiesteban C (2014) Optical coherence tomography shows retinal abnormalities associated with optic nerve disease. Br J Ophthalmol 98: ii30–3. 24627251 10.1136/bjophthalmol-2013-304301 PMC4208346

[20] You Y, Gupta VK, Li JC, (2013) Optic neuropathies: characteristic features and mechanisms of retinal ganglion cell loss. Rev Neurosci 24: 301–321. 23612594 10.1515/revneuro-2013-0003

[21] Namekata K, Kimura A, Kawamura K, (2013) Dock3 attenuates neural cell death due to NMDA neurotoxicity and oxidative stress in a mouse model of normal tension glaucoma. Cell Death Diff 20: 1250–1256. 10.1038/cdd.2013.91 PMC3741514 23852370

[22] Burkholder BM, Osborne B, Loguidice MJ, (2009) Macular volume determined by optical coherence tomography as a measure of neuronal loss in multiple sclerosis. Arch Neurol 66: 1366. 19901168 10.1001/archneurol.2009.230

[23] Frohman EM, Dwyer MG, Frohman T, (2009) Relationship of optic nerve and brain conventional and non-conventional MRI measures and retinal nerve fiber layer thickness, as assessed by OCT and GDx: a pilot study. J Neurol Sci 282: 96–105. 19439327 10.1016/j.jns.2009.04.010

[24] Pineles SL, Birch EE, Talman LS, (2011) One eye or two: a comparison of binocular and monocular low-contrast acuity testing in multiple sclerosis. Am J Ophthalmol 152: 133–140. 21570055 10.1016/j.ajo.2011.01.023 PMC3637955

[25] Martin R, Mcfarland HF, Mcfarlin DE (1992) Immunological aspects of demyelinating diseases. Annu Rev Immunol 10: 153. 1375472 10.1146/annurev.iy.10.040192.001101

[26] Hobom M, Storch MK, Weissert R, (2004) Mechanisms and time course of neuronal degeneration in experimental autoimmune encephalomyelitis. Brain Pathol 14: 148–157. 15193027 10.1111/j.1750-3639.2004.tb00047.x PMC8095969

[27] Croxford AL, Kurschus FC, Waisman A (2011) Mouse models for multiple sclerosis: historical facts and future implications. Biochim Biophys Acta 1812: 177–183. 20600870 10.1016/j.bbadis.2010.06.010

[28] Shields DC, Banik NL (1999) Pathophysiological role of calpain in experimental demyelination. J Neurosci Res 55: 533–541. 10082076 10.1002/(SICI)1097-4547(19990301)55:5<533::AID-JNR1>3.0.CO;2-8

[29] Hassen GW, Feliberti J, Kesner L, (2006) A novel calpain inhibitor for the treatment of acute experimental autoimmune encephalomyelitis. J Neuroimmunol 180: 135–146. 17007940 10.1016/j.jneuroim.2006.08.005

[30] Guyton MK, Das A, Samantaray S, (2010) Calpeptin attenuated inflammation, cell death, and axonal damage in animal model of multiple sclerosis. J Neurosci Res 88: 2398–2408. 20623621 10.1002/jnr.22408 PMC3164817

[31] Kobeissy FH. (2015) Brain Neurotrauma: Molecular, Neuropsychological, and Rehabilitation Aspects. Crc Press. 26269865

[32] Sun JF, Yang HL, Huang YH, (2017) CaSR and calpain contribute to the ischemia reperfusion injury of spinal cord. Neurosci lett 646: 49–55. 28284837 10.1016/j.neulet.2017.03.009

[33] Samantaray S, Knaryan VH, Shields DC, (2013) Critical role of calpain in spinal cord degeneration in Parkinson’s disease. J Neurochem 127: 880. 23875735 10.1111/jnc.12374 PMC3859802

[34] Siklos M, Benaissa M, Thatcher GRJ (2015) Cysteine proteases as therapeutic targets: does selectivity matter? A systematic review of calpain and cathepsin inhibitors. Acta pharmaceutica Sinica B 5: 506–519. 26713267 10.1016/j.apsb.2015.08.001 PMC4675809

[35] Smith AW, Rohrer B, Wheless L, (2016) Calpain inhibition reduces structural and functional impairment of retinal ganglion cells in experimental optic neuritis. J Neurochem 139: 270–284. 27513991 10.1111/jnc.13770 PMC5056830

[36] Ono Y, Sorimachi H (2012) Calpains: an elaborate proteolytic system. Biochim Biophys Acta 1824: 224–236. 21864727 10.1016/j.bbapap.2011.08.005

[37] Pietrobon D, Di VF, Pozzan T (1990) Structural and functional aspects of calcium homeostasis in eukaryotic cells. Eur J Biochem 193: 599–622. 2249682 10.1111/j.1432-1033.1990.tb19378.x

[38] Wu HY, Tomizawa K, Matsui H (2007) Calpain-calcineurin signaling in the pathogenesis of calcium-dependent disorder. Acta Medica Okayama 61: 123–137. 17593948 10.18926/AMO/32905

[39] Diaz-Sanchez M, Williams K, Deluca GC, (2006) Protein co-expression with axonal injury in multiple sclerosis plaques. Acta Neuropathol 111: 289. 16547760 10.1007/s00401-006-0045-0

[40] Yan F, Sun W, Shi Y, (2009) Glutamate excitotoxicity inflicts paranodal myelin splitting and retraction. Plos One 4: e6705. 19693274 10.1371/journal.pone.0006705 PMC2725320

[41] Banik NL, Chou CH, Deibler GE, (1994) Peptide bond specificity of calpain: proteolysis of human myelin basic protein. J Neurosci Res 37: 489–496. 7517457 10.1002/jnr.490370408

[42] Howe CL, Lafrancecorey RG, Mirchia K, (2016) Neuroprotection mediated by inhibition of calpain during acute viral encephalitis. Sci Rep 6: 28699. 27345730 10.1038/srep28699 PMC4921808

[43] Machado VM, Morte MI, Carreira BP, (2015) Involvement of calpains in adult neurogenesis: implications for stroke. Front Cell Neurosci 9: 22. 25698931 10.3389/fncel.2015.00022 PMC4316774

[44] Knaryan VH, Samantaray S, Park S, (2014) SNJ-1945, a calpain inhibitor, protects SH-SY5Y cells against MPP(+) and rotenone. J Neurochem 130: 280–290. 24341912 10.1111/jnc.12629 PMC4038676

[45] Donkor IO (2000) A survey of calpain inhibitors. Curr Med Chem 7: 1171–1181. 11032966 10.2174/0929867003374129

[46] Shirasaki Y, Miyashita H, Yamaguchi M, (2005) Exploration of orally available calpain inhibitors: peptidyl alpha-ketoamides containing an amphiphile at P3 site. Bioorg Med Chem 13: 4473–4484. 15921914 10.1016/j.bmc.2005.04.059

[47] Donkor IO, Zheng X, Miller DD (2000) Synthesis and calpain inhibitory activity of alpha-ketoamides with 2,3-methanoleucine stereoisomers at the P2 position. Bioorg Med Chem Lett 10: 2497–2500. 11086714 10.1016/s0960-894x(00)00518-7

[48] Carragher NO (2006) Calpain inhibition: a therapeutic strategy targeting multiple disease states. Curr Pharm Design 12: 615–638. 10.2174/138161206775474314 16472152

[49] Barrett AJ, Kembhavi AA, Brown MA, (1982) L-trans-Epoxysuccinyl-leucylamido (4-guanidino) butane (E-64) and its analogues as inhibitors of cysteine proteinases including cathepsins B, H and L. Biochem J 201: 189–198. 7044372 10.1042/bj2010189 PMC1163625

[50] Saido TC, Sorimachi H, Suzuki K (1994) Calpain: new perspectives in molecular diversity and physiological-pathological involvement. FASEB J 8: 814–822. 8070630

[51] Podbielska M, Das A, Smith AW, (2016) Neuron-microglia interaction induced bi-directional cytotoxicity associated with calpain activation. J Neurochem 139: 440–455. 27529445 10.1111/jnc.13774 PMC5374727

[52] Xiao T, Zhang Y, Wang Y, (2013) Activation of an apoptotic signal transduction pathway involved in the upregulation of calpain and apoptosis-inducing factor in aldosterone-induced primary cultured cardiomyocytes. Food Chem Toxicol 53: 364–370. 23266505 10.1016/j.fct.2012.12.022

[53] Imai T, Kosuge Y, Endo-Umeda K, (2014) Protective effect of S-allyl-L-cysteine against endoplasmic reticulum stress-induced neuronal death is mediated by inhibition of calpain. Amino Acids 46: 385–393. 24287800 10.1007/s00726-013-1628-4

[54] Li H, Zhang N, Sun G, (2013) Inhibition of the group I mGluRs reduces acute brain damage and improves long-term histological outcomes after photothrombosis-induced ischaemia. ASN Neuro 5: 195. 23772679 10.1042/AN20130002 PMC3786425

[55] Ray SK, Neuberger TJ, Deadwyler G, (2002) Calpain and calpastatin expression in primary oligodendrocyte culture: preferential localization of membrane calpain in cell processes. J Neurosci Res 70: 561–569. 12404510 10.1002/jnr.10414

[56] Das A, Garner DP, Del Re AM, (2006) Calpeptin provides functional neuroprotection to rat retinal ganglion cells following Ca2+ influx. Brain Res 1084: 146–157. 16600192 10.1016/j.brainres.2006.02.051

[57] Guyton MK, Brahmachari S, Das A, (2009) Inhibition of calpain attenuates encephalitogenicity of MBP-specific T cells. J Neurochem 110: 1895–1907. 19627443 10.1111/j.1471-4159.2009.06287.x PMC2748265

[58] Guyton MK, Das A, Samantaray S, (2010) Calpeptin attenuated inflammation, cell death, and axonal damage in animal model of multiple sclerosis. J Neurosci Res 88: 2398–2408. 20623621 10.1002/jnr.22408 PMC3164817

[59] Neffe AT, Abell AD (2005) Developments in the design and synthesis of calpain inhibitors. Curr Opin Drug Discov Develop 8: 684–700. 16312145

[60] Das A, Sribnick EA, Wingrave JM, (2005) Calpain activation in apoptosis of ventral spinal cord 4.1 (VSC4.1) motoneurons exposed to glutamate: calpain inhibition provides functional neuroprotection. J Neurosci Res 81: 551–562. 15968645 10.1002/jnr.20581

[61] Sharma A1 (2007) Sustained elevation of intracellular cGMP causes oxidative stress triggering calpain-mediated apoptosis in photoreceptor degeneration. Curr Eye Res 32: 259–269. 17453946 10.1080/02713680601161238

[62] Min Y, Zhu W, Zheng X, (2016) Effect of glutamate on lysosomal membrane permeabilization in primary cultured cortical neurons. Mol Med Rep 13: 2499–2505. 26821268 10.3892/mmr.2016.4819 PMC4768955

[63] Shirasaki Y, Yamaguchi MH (2006) Retinal penetration of calpain inhibitors in rats after oral administration. J Ocul Pharmacol Ther 22: 417–424. 17238807 10.1089/jop.2006.22.417

[64] Koumura A, Nonaka Y, Hyakkoku K, (2008). A novel calpain inhibitor, ((1S)-1((((1S)-1-benzyl-3-cyclopropylamino-2,3-di-oxopropyl)amino)carbonyl)-3-met hylbutyl) carbamic acid 5-methoxy-3-oxapentyl ester, protects neuronal cells from cerebral ischemia-induced damage in mice. Neurosci 157: 309–318. 10.1016/j.neuroscience.2008.09.007 18835333

[65] Nakajima E, Hammond KB, Rosales JL, (2011) Calpain, not caspase, is the causative protease for hypoxic damage in cultured monkey retinal cells. Invest Ophthalmol Vis Sci 52: 7059–7067. 21757584 10.1167/iovs.11-7497 PMC3207712

[66] Trager N, Butler JT, Haque A, (2013) The Involvement of Calpain in CD4+ T Helper Cell Bias in Multple Sclerosis. J Clin Cell Iimmunol 4: 1000153. 10.4172/2155-9899.1000153 PMC3972924 24707444

[67] Surh YJ, Chun KS, Cha HH, (2001) Molecular mechanisms underlying chemopreventive activities of anti-inflammatory phytochemicals: down-regulation of COX-2 and iNOS through suppression of NF-kappa B activation. Mutat Res s480–481: 243–268. 10.1016/s0027-5107(01)00183-x 11506818

[68] Potz BA, Sabe AA, Elmadhun NY, (2017) Calpain inhibition decreases inflammatory protein expression in vessel walls in a model of chronic myocardial ischemia. Surgery 161: 1394–1404. 28024857 10.1016/j.surg.2016.11.009 PMC5404979

[69] Butler JT, Samantaray S, Beeson CC, (2009) Involvement of calpain in the process of Jurkat T cell chemotaxis. J Neurosci Res 87: 626–635. 18831007 10.1002/jnr.21882 PMC2678561

[70] Mikosik A, Jasiulewicz A, Daca A, (2016) Roles of calpain-calpastatin system (CCS) in human T cell activation. Oncotarget 7: 76479–76495. 27835610 10.18632/oncotarget.13259 PMC5363525

[71] Moretti D, Del BB, Allavena G, (2014) Calpains and cancer: friends or enemies? Arch Biochem Biophys 564: 26–36. 25305531 10.1016/j.abb.2014.09.018

[72] Deshpande RV, Goust JM, Hogan EL, (1995) Calpain secreted by activated human lymphoid cells degrades myelin. J Neurosci Res 42: 259–265. 8568927 10.1002/jnr.490420214

[73] Jantzie LL, Winer JL, Corbett CJ, (2016) Erythropoietin Modulates Cerebral and Serum Degradation Products from Excess Calpain Activation following Prenatal Hypoxia-Ischemia. Dev Neurosci 38: 15–26. 26551007 10.1159/000441024 PMC4732905

[74] Guyton MK, Sribnick EA, Ray SK, (2005) A role for calpain in optic neuritis. Ann NY Acad Sci 1053: 48–54. 16179508 10.1196/annals.1344.005

[75] Cerghet M, Skoff RP, Bessert D, (2006) Proliferation and death of oligodendrocytes and myelin proteins are differentially regulated in male and female rodents. J Neurosci 26: 1439–1447. 16452667 10.1523/JNEUROSCI.2219-05.2006 PMC6675481

[76] Ding ZJ, Chen X, Tang XX, (2015) Calpain inhibitor PD150606 attenuates glutamate induced spiral ganglion neuron apoptosis through apoptosis inducing factor pathway in vitro. PLoS One 10: e0123130. 25874633 10.1371/journal.pone.0123130 PMC4398365

[77] Das A, Guyton MK, Matzelle DD, (2008) Time-dependent increases in protease activities for neuronal apoptosis in spinal cords of Lewis rats during development of acute experimental autoimmune encephalomyelitis. J Neurosci Res 86: 2992–3001. 18521931 10.1002/jnr.21737 PMC2614291

[78] Vosler PS, Brennan CS, Chen J (2008) Calpain-mediated signaling mechanisms in neuronal injury and neurodegeneration. Mol Neurobiol 38: 78–100. 18686046 10.1007/s12035-008-8036-x PMC2726710

[79] Schaecher K, Goust JM, Banik NL (2004) The effects of calpain inhibition on IkB alpha degradation after activation of PBMCs: identification of the calpain cleavage sites. Neurochem Res 29: 1443–1451. 15202778 10.1023/b:nere.0000026410.56000.dd

[80] Gerondakis S, Grumont R, Rourke I, (1998) The regulation and roles of Rel/NF-kappa B transcription factors during lymphocyte activation. Curr Opin Immunol 10: 353–359. 9638373 10.1016/s0952-7915(98)80175-1

[81] Miyazaki T, Taketomi Y, Saito Y, (2015) Calpastatin counteracts pathological angiogenesis by inhibiting suppressor of cytokine signaling 3 degradation in vascular endothelial cells. Circ Res 116: 1170–1181. 25648699 10.1161/CIRCRESAHA.116.305363

[82] Datta R, Naura AS, Zerfaoui M, (2011) PARP-1 deficiency blocks IL-5 expression through calpain-dependent degradation of STAT-6 in a murine asthma model. Allergy 66: 853–861. 21276008 10.1111/j.1398-9995.2011.02549.x PMC3150522

[83] Tahvanainen J, Kallonen T, Lähteenmäki H, (2009) PRELI is a mitochondrial regulator of human primary T-helper cell apoptosis, STAT6, and Th2-cell differentiation. Blood 113: 1268–1277. 18945965 10.1182/blood-2008-07-166553

[84] Hendry L, John S (2004) Regulation of STAT signalling by proteolytic processing. Eur J Biochem 271: 4613–4620. 15606748 10.1111/j.1432-1033.2004.04424.x

[85] Svensson L, Mcdowall A, Giles KM, (2010) Calpain 2 controls turnover of LFA-1 adhesions on migrating T lymphocytes. PLoS One 5: e15090. 21152086 10.1371/journal.pone.0015090 PMC2994845

[86] Stewart MP, Mcdowall A, Hogg N (1998) LFA-1-mediated adhesion is regulated by cytoskeletal restraint and by a Ca2+-dependent protease, calpain. J cell Biol 140: 699–707. 9456328 10.1083/jcb.140.3.699 PMC2140165

[87] Soede RD, Driessens MH, Ruulsvan SL, (1999) LFA-1 to LFA-1 signals involve zeta-associated protein-70 (ZAP-70) tyrosine kinase: relevance for invasion and migration of a T cell hybridoma. J Immunol 163: 4253–4261. 10510363

[88] Engelhard VH, Altrich-Vanlith M, Ostankovitch M, (2006) Post-translational modifications of naturally processed MHC-binding epitopes. Curr Opin Immunol 18: 92–97. 16343885 10.1016/j.coi.2005.11.015

[89] Imam SA, Guyton MK, Haque A, (2007) Increased calpain correlates with Th1 cytokine profile in PBMCs from MS patients. J Neuroimmunol 190: 139–145. 17765980 10.1016/j.jneuroim.2007.07.016 PMC2096747

[90] Zhang Z, Huang Z, Dai H, (2015) Therapeutic Efficacy of E-64-d, a Selective Calpain Inhibitor, in Experimental Acute Spinal Cord Injury. BioMed Res Int: 134242. 26240815 10.1155/2015/134242 PMC4512559

[91] Posmantur R, Hayes RL, Dixon CE, (1994) Neurofilament 68 and neurofilament 200 protein levels decrease after traumatic brain injury. J Neurotrauma 11: 533–545. 7861446 10.1089/neu.1994.11.533

[92] Zhang JN, Michel U, Lenz C, (2016) Calpain-mediated cleavage of collapsin response mediator protein-2 drives acute axonal degeneration. Sci Rep 6: 37050. 27845394 10.1038/srep37050 PMC5109185

[93] Wilson GN, Smith MA, Inman DM, (2016) Early Cytoskeletal Protein Modifications Precede Overt Structural Degeneration in the DBA/2J Mouse Model of Glaucoma. Front Neurosci 10: 494. 27857681 10.3389/fnins.2016.00494 PMC5093131

[94] Saito K, Elce JS, Hamos E, (1993) Widespread activation of calcium-activated neutral proteinase (calpain) in the brain in Alzheimer disease: a potential molecular basis for neuronal degeneration. Proc Natl Acad Sci USA 90: 2628–2632. 8464868 10.1073/pnas.90.7.2628 PMC46148

[95] Dowling P, Husar W, Menonna J, (1997) Cell death and birth in multiple sclerosis brain. J Neurol Sci 149: 1–11. 9168159 10.1016/s0022-510x(97)05213-1

[96] Gerónimoolvera C, Montiel T, Rinconheredia R, (2017) Autophagy fails to prevent glucose deprivation/glucose reintroduction-induced neuronal death due to calpain-mediated lysosomal dysfunction in cortical neurons. Cell Death Disease 8: e2911. 28661473 10.1038/cddis.2017.299 PMC5520945

[97] Sharma AK, Rohrer B (2017) Calcium-induced calpain mediates apoptosis via caspase-3 in a mouse photoreceptor cell line. J Biol Chem 292: 13186. 28801351 10.1074/jbc.A117.401037 PMC5555181

[98] Pitt D, Werner P, Raine CS (2000) Glutamate excitotoxicity in a model of multiple sclerosis. Nat Med 6: 67–70. 10613826 10.1038/71555

[99] Shields DC, Banik NL (1999) A putative mechanism of demyelination in multiple sclerosis by a proteolytic enzyme, calpain. Proc Natl Acad Sci USA 96: 11486–11491. 10500203 10.1073/pnas.96.20.11486 PMC18060

[100] Zhang ZW, Qin XY, Che FY, (2015) Effects of beta 2 adrenergic agonists on axonal injury and mitochondrial metabolism in experimental autoimmune encephalomyelitis rats. Genetics and molecular research: GMR 14: 13572–13581. 26535670 10.4238/2015.October.28.17

[101] Kivisäkk P, Mahad DJ, Callahan MK, (2003) Human cerebrospinal fluid central memory CD4+ T cells: evidence for trafficking through choroid plexus and meninges via P-selectin. Proc Natl Acad Sci USA 100: 8389–8394. 12829791 10.1073/pnas.1433000100 PMC166239

[102] Lassmann H, Ransohoff RM (2004) The CD4-Th1 model for multiple sclerosis: a critical [correction of crucial] re-appraisal. Trends Immunol 25: 132–137. 15036040 10.1016/j.it.2004.01.007

[103] Dhib S (2003) Glatiramer acetate-reactive peripheral blood mononuclear cells respond to multiple myelin antigens with a Th2-biased phenotype. J Neuroimmunol 140: 163–171. 12864985 10.1016/s0165-5728(03)00170-x

[104] Issazadeh S, Mustafa M, Ljungdahl A, (1995) Interferon gamma, interleukin 4 and transforming growth factor beta in experimental autoimmune encephalomyelitis in Lewis rats: dynamics of cellular mRNA expression in the central nervous system and lymphoid cells. J Neurosci Res 40: 579–590. 7602612 10.1002/jnr.490400503

[105] Kennedy MK, Torrance DS, Picha KS, (1992) Analysis of cytokine mRNA expression in the central nervous system of mice with experimental autoimmune encephalomyelitis reveals that IL-10 mRNA expression correlates with recovery. J Immunol 149: 2496. 1527389

[106] Nishihara M, Ogura H, Ueda N, (2007) IL-6-gp130-STAT3 in T cells directs the development of IL-17+ Th with a minimum effect on that of Treg in the steady state. Int Immunol 19: 695–702. 17493959 10.1093/intimm/dxm045

[107] Immunology N (2005) Interleukin 17-producing CD4+ effector T cells develop via a lineage distinct from the T helper type 1 and 2 lineages. Nat Immunol 6: 1123–1132. 16200070 10.1038/ni1254

[108] Park H, Li Z, Yang XO, (2005) A distinct lineage of CD4 T cells regulates tissue inflammation by producing interleukin 17. Nat Immunol 6: 1133–1141. 16200068 10.1038/ni1261 PMC1618871

[109] Passos GRD, Sato DK, Becker J, (2016) Th17 Cells Pathways in Multiple Sclerosis and Neuromyelitis Optica Spectrum Disorders: Pathophysiological and Therapeutic Implications. Mediat Inflamm 2016: 1–11. 10.1155/2016/5314541 PMC4749822 26941483

[110] Zahra S, Rozita D, Masoumeh B, (2016) Differential Frequency of CD8+ T Cell Subsets in Multiple Sclerosis Patients with Various Clinical Patterns. PLoS One 11: e0159565. 27467597 10.1371/journal.pone.0159565 PMC4965085

[111] Kebir H, Ifergan I, Alvarez JI, (2009) Preferential recruitment of interferon-gamma-expressing TH17 cells in multiple sclerosis. Ann Neurol 66: 390–402. 19810097 10.1002/ana.21748

[112] Thackray SJ, Mowat CG, Chapman SK (2008) Exploring the mechanism of tryptophan 2,3-dioxygenase. Biochem Soc Trans 36: 1120–1123. 19021508 10.1042/BST0361120 PMC2652831

[113] Stone TW, Darlington LG (2002) Endogenous kynurenines as targets for drug discovery and development. Nat Rev 1: 609–620. 10.1038/nrd870 12402501

[114] Munn DH, Mellor AL (2007) Indoleamine 2,3-dioxygenase and tumor-induced tolerance. J Clin Invest 117: 1147–1154. 17476344 10.1172/JCI31178 PMC1857253

[115] Fallarino F, Grohmann U, You S, (2006) The combined effects of tryptophan starvation and tryptophan catabolites down-regulate T cell receptor zeta-chain and induce a regulatory phenotype in naive T cells. J Immunol 176: 6752–6761. 16709834 10.4049/jimmunol.176.11.6752

[116] Mackenzie CR, Heseler K, Müller A, (2007) Role of indoleamine 2,3-dioxygenase in antimicrobial defence and immuno-regulation: tryptophan depletion versus production of toxic kynurenines. Curr Drug Metab 8: 237–244. 17430112 10.2174/138920007780362518

[117] Mancuso R, Hernis A, Agostini S, (2015) Indoleamine 2,3 Dioxygenase (IDO) Expression and Activity in Relapsing-Remitting Multiple Sclerosis. Plos One 10: e0130715. 26110930 10.1371/journal.pone.0130715 PMC4482492

[118] Sousa DA, Porto WF, Silva MZ, (2016) Influence of Cysteine and Tryptophan Substitution on DNA-Binding Activity on Maize Î±-Hairpinin Antimicrobial Peptide. Molecules 21: 1062. 27529210 10.3390/molecules21081062 PMC6273665

[119] Smith AW, Doonan BP, Tyor WR, (2011) Regulation of Th1/Th17 cytokines and IDO gene expression by inhibition of calpain in PBMCs from MS patients. J Neuroimmunol 232: 179–185. 21075457 10.1016/j.jneuroim.2010.09.030 PMC3053080

[120] Cilingir V, Batur M, Bulut MD, (2017) The association between retinal nerve fibre layer thickness and corpus callosum index in different clinical subtypes of multiple sclerosis. Neurol Sci 38: 1223–1232. 28396954 10.1007/s10072-017-2947-0

[121] Britze J, Pihljensen G, Frederiksen JL (2017) Retinal ganglion cell analysis in multiple sclerosis and optic neuritis: a systematic review and meta-analysis. J Neurol 264: 1837–1853. 28567539 10.1007/s00415-017-8531-y

[122] Mowry EM, Loguidice MJ, Daniels AB, (2009) Vision related quality of life in multiple sclerosis: correlation with new measures of low and high contrast letter acuity. J Neurol Neurosurg Psychia 80: 767–772. 10.1136/jnnp.2008.165449 19240050

[123] Mackay DD (2015) Should patients with optic neuritis be treated with steroids? Curr Opin Ophthalmol 26: 439–444. 26367086 10.1097/ICU.0000000000000197

[124] Dimitriu C, Bach M, Lagrèze WA, (2008) Methylprednisolone fails to preserve retinal ganglion cells and visual function after ocular ischemia in rats. Invest Ophthalmol Vis Sci 49: 5003–5007. 18586870 10.1167/iovs.08-1869

[125] Das A, Guyton MK, Smith A, (2013) Calpain inhibitor attenuated optic nerve damage in acute optic neuritis in rats. J Neurochem 124: 133–143. 23106593 10.1111/jnc.12064 PMC3570118

[126] Shields DC, Tyor WR, Deibler GE, (1998) Increased calpain expression in experimental demyelinating optic neuritis: an immunocytochemical study. Brain Res 784: 299–304. 9518658 10.1016/s0006-8993(97)01381-4

[127] Hoffmann DB, Williams SK, Jovana B, (2013) Calcium influx and calpain activation mediate preclinical retinal neurodegeneration in autoimmune optic neuritis. J Neuropathol Exp Neurol 72: 745–757. 23860028 10.1097/NEN.0b013e31829c7370

[128] Shimazawa M, Suemori S, Inokuchi Y, (2010) A novel calpain inhibitor, ((1S)-1-((((1S)-1-Benzyl-3-cyclopropylamino-2,3-di-oxopropyl)amino)carbonyl)-3-met hylbutyl)carbamic acid 5-methoxy-3-oxapentyl ester (SNJ-1945), reduces murine retinal cell death in vitro and in vivo. J Pharm Exp Ther 332: 380–387. 10.1124/jpet.109.156612 19910537

[129] Imai S, Shimazawa M, Nakanishi T, (2010) Calpain inhibitor protects cells against light-induced retinal degeneration. J Pharm Exp Ther 335: 645–652. 10.1124/jpet.110.171298 20823194

[130] Yokoyama Y, Maruyama K, Yamamoto K, (2014) The role of calpain in an in vivo model of oxidative stress-induced retinal ganglion cell damage. Biochem Biophys Res Comm 451: 510–515. 25111816 10.1016/j.bbrc.2014.08.009

[131] Wang Y, Liu Y, Lopez D, (2017) Protection against TBI-induced neuronal death with post-treatment with a selective calpain-2 inhibitor in mice. J Neurotrauma. 10.1089/neu.2017.5024 PMC5757088 28594313

[132] Tsuda S, Tanaka Y, Kunikata H, (2016) Real-time imaging of RGC death with a cell-impermeable nucleic acid dyeing compound after optic nerve crush in a murine model. Exp Eye Res 146: 179–188. 27013099 10.1016/j.exer.2016.03.017

[133] Ryu M, Yasuda M, Shi D, (2012) Critical role of calpain in axonal damage-induced retinal ganglion cell death. J Neurosci Res 90: 802–815. 22065590 10.1002/jnr.22800

[134] Nemeth CL, Drummond GT, Mishra MK, (2017) Uptake of dendrimer-drug by different cell types in the hippocampus after hypoxic-ischemic insult in neonatal mice: Effects of injury, microglial activation and hypothermia. Nanomedicine 13: 2359–2369. 28669854 10.1016/j.nano.2017.06.014 PMC5849407

[135] Zhang F, Trent MJ, Lin YA, (2017) Generation-6 hydroxyl PAMAM dendrimers improve CNS penetration from intravenous administration in a large animal brain injury model. J Control Release 249: 173–182. 28137632 10.1016/j.jconrel.2017.01.032 PMC5323327

[136] Balcer LJ, Galetta SL, Calabresi PA, (2007) Natalizumab reduces visual loss in patients with relapsing multiple sclerosis. Neurology 68: 1299–1304. 17438220 10.1212/01.wnl.0000259521.14704.a8

[137] Sättler MB, Demmer I, Williams SK, (2006) Effects of interferon-beta-1a on neuronal survival under autoimmune inflammatory conditions. Exp Neurol 201: 172–181. 16764858 10.1016/j.expneurol.2006.04.015

[138] Coleman M (2005) Axon degeneration mechanisms: commonality amid diversity. Nat Rev Neurosci 6: 889–898. 16224497 10.1038/nrn1788

